# Modeling growth, development and yield of cassava: A review

**DOI:** 10.1016/j.fcr.2021.108140

**Published:** 2021-06-15

**Authors:** Patricia Moreno-Cadena, Gerrit Hoogenboom, James H. Cock, Julian Ramirez-Villegas, Pieter Pypers, Christine Kreye, Meklit Tariku, Kodjovi Senam Ezui, Luis Augusto Becerra Lopez-Lavalle, Senthold Asseng

**Affiliations:** a Agricultural and Biological Engineering Department, University of Florida, 101 Frazier Rogers Hall, PO Box 110570, Gainesville, FL, 32611-0570, USA; b Alliance of Bioversity International and International Center for Tropical Agriculture (CIAT), km 17 recta Cali–Palmira, 763537, Cali, Colombia; c International Institute of Tropical Agriculture (IITA), Ibadan, Nigeria; d Institute for Sustainable Food Systems, University of Florida, 101 Frazier Rogers Hall, PO Box 110570, Gainesville, FL, 32611-0570, USA; e CGIAR Research Program on Climate Change, Agriculture and Food Security (CCAFS), Cali, Colombia; f International Institute of Tropical Agriculture (IITA), ICIPE Campus, P.O. Box 30772-00100, Nairobi, Kenya; g African Plant Nutrition Institute (APNI), ICIPE Campus, Duduville – Kasarani, Thika Road, Nairobi, Kenya

**Keywords:** Dry matter content, Storage roots, Crop simulation models, Decision support systems, Food security

## Abstract

Cassava is an important crop in the developing world. The goal of this study was to review published cassava models (18) for their capability to simulate storage root biomass and to categorize them into static and dynamic models. The majority (14) are dynamic and capture within season growth dynamics. Most (13) of the dynamic models consider environmental factors such as temperature, solar radiation, soil water and nutrient restrictions. More than half (10) have been calibrated for a distinct genotype. Only one of the four static models includes environmental variables. While the static regression models are useful to estimate final yield, their application is limited to the locations or varieties used for their development unless recalibrated for distinct conditions. Dynamic models simulate growth process and provide estimates of yield over time with, in most cases, no fixed maturity date. The dynamic models that simulate the detailed development of nodal units tend to be less accurate in determining final yield compared to the simpler dynamic and statistic models. However, they can be more safely applied to novel environmental conditions that can be explored *in silico.* Deficiencies in the current models are highlighted including suggestions on how they can be improved. None of the current dynamic cassava models adequately simulates the starch content of fresh cassava roots with almost all models based on dry biomass simulations. Further studies are necessary to develop a new module for existing cassava models to simulate cassava quality.

## Highlights

• The majority (14) of the reviewed cassava models are dynamic.
• Detailed dynamic models tend to be less accurate in determining final yield.
• Only one of the four static models includes environmental variables.
• Cassava models do not represent the dynamics of starch content in fresh weight.

## 1 Introduction

Cassava (*Manihot esculenta* Crantz) is the fourth most important source of calories in Africa (FAO, 2020). For some countries of Africa, approximately 25 % of the daily calorie intake is provided by cassava (De Souza et al., 2016). Although cassava has long been a staple crop for most of the countries in Africa, it is increasingly considered as a cash crop (Nweke, 2005). It is also a major source of starch with highly industrialized extraction in countries like Vietnam and Thailand in Asia (Howeler, 2012) and Brazil and Paraguay in the Americas (Aristizabal Galvis et al., 2017).

Crop models have shown to be valuable tools to predict growth and development and inform agronomic interventions (Tsuji et al., 1998). However, cassava has been less well studied compared to other crops despite its importance as a food in many of the developing countries (Leal et al., 2014; Varshney et al., 2010) and its commercial use for starch in the tropics and subtropics (Karlström et al., 2016; Nweke, 2005). Surprisingly, the number of cassava simulation models that have been published is close to 20. The first report of a cassava model was from the International Center for Tropical Agriculture (CIAT) (CIAT, 1976). At about the same time Boerboom (1978) defined a statistical linear relationship between total biomass accumulation and yield after a threshold of total plant biomass was reached. The most recently published model is a dynamic process-based model (Connor, 2019).

The overall goal of this study was to gain new insights from the literature and ongoing development to provide the background for improving the simulation of cassava growth and development as a function of genetics, crop management and environmental factors. This study reviews existing cassava models that simulate dry biomass production and estimate tuber yield. When the model developers do not provide a name for the model, we use the name of the first author of the original paper, or both authors when there are only two.

## 2 Overview of cassava growth models

In this overview, the major features of various cassava models are presented and related to the overall structure and development of the cassava crop. Later, in the section that describes the individual models (section 3), details are provided on how each model functions, highlighting their major features and novel approaches. To make this review easier to read we have roughly classified the models as follows.

• Dynamic models that describe the growth and development of the plant over time.
• Static or regression-based models that provide an estimate of the expected final yield at final harvest but do not consider how that yield develops over time.

### 2.1 Dynamic models

The dynamic models vary from simple models that describe the overall growth and development of a single cultivar under a specific set of conditions to those that consider variation in weather, soil, crop management and variety. All dynamic models include partial differential equations that function with time steps, ranging from weekly to hourly in the latest models. In most of these models the resources accumulated during period *i* becomes available for growth in the following period *i+1*.

The first dynamic model, the Cock Model, defined the major crop features that describe crop development in dynamic models (Cock et al., 1979). These are: (i) the development of leaf area over time; (ii) the relation between the production of biomass and leaf area; and (iii) distribution of biomass and new growth to the distinct organs of the plant. The next major advance in cassava modeling was the dynamic Fukai-Hammer model (Fukai and Hammer, 1987), which incorporated variation in weather, soil fertility, and soil conditions. They also implemented the concept of thermal time with a base temperature which became standard procedure in the subsequent dynamic models. The dynamic models that were developed later refine these basic processes. In the following sections, we highlight the range of approaches used to simulate these processes.

#### 2.1.1 Plant structure

Cassava has no well-defined growth stages such as anthesis, nor does it reach physiological maturity and thus harvest time under field conditions varies greatly depending on farmer management. Many of the models use arbitrarily determined growth stages, but there is no general agreement on these divisions. Some models treat the crop as having a fixed harvest date or time to maturity; however, most dynamic models allow cassava to continue growing. The dynamic models consider the overall structure of the plant with varying levels of detail but with the major components of leaves, stems, fibrous or feeder roots and the tubers (Cock et al., 1979; Fukai and Hammer, 1987). Several of the more detailed dynamic models are driven by the development of cohorts of individual phytomers, the nodal units, whilst others simulate the dynamic balance between distinct organs.

#### 2.1.2 Development of leaf area

The effective leaf area in the dynamic models is the net result of both leaf formation and leaf shedding following senescence. The formation of leaf area derives either from the development of the cohorts of nodes or from generalized patterns of overall leaf area formation and the distribution of biomass between organs. The potential growth of new leaf area is normally restricted by the amount of available assimilate (see below sections on biomass accumulation (2.1.3) and distribution (2.1.4)).

#### 2.1.3 Biomass accumulation

Dynamic models determine biomass assimilation via a range of approaches from a simple relationship of crop growth rate to leaf area index (LAI), estimates of intercepted PAR and radiation use efficiency (RUE), to estimation of photosynthesis considering not only leaf area but also canopy structure, respiration losses and the cost of biosynthesis.

#### 2.1.4 Biomass partition

Most crop models use empirically determined factors to define the distribution of biomass to the various organs based on distinct growth phases. Two principal approaches have been used to model the partitioning of biomass in cassava: (i) empirically determined factors which define the fraction of biomass accumulation in the various plant parts, and (ii) the spillover model. The use of empirically determined factors was first proposed by Boerboom (1978), who used a constant distribution of biomass to the storage roots after a threshold total plant weight was reached. Several of the cassava models use variations on this approach. In contrast, the spill-over model gives preference for assimilate allocation to top growth and only when the available assimilate is greater than the demand of the tops it spills over into the tubers.

Some of the dynamic models allow for the accumulation of labile assimilate reserves, which can be relocated to other plant organs. This allows for simulating flushes of new top growth when it rains after a long dry period. Similarly, several of the models also allow translocation of the nutrients and assimilates from senescing leaves.

#### 2.1.5 Drivers of the models

The main drivers of the crop response include solar radiation, air temperature, rainfall, vapor pressure deficit (VPD), plant nutrients, photoperiod, and CO**_2_** concentration. Solar radiation acts in the models as a driver of carbon assimilation, whereas temperature affects both the assimilation rates and crop development. Below a base temperature, i.e., 10−16 °C depending on the model, crop development ceases. Crop development is also affected by photoperiod, with long days promoting flowering. Rainfall effects are principally through plant extractable soil moisture. As the soil water content decreases stomatal conductance is reduced emulating stomatal closure, which reduces carbon assimilation and transpiration as well as nutrient uptake. Where included, VPD is often calculated within models using psychrometric equations and with reduced stomatal conductance or radiation use efficiency at high VPD values.

### 2.2 Static or regression-based models

The static models do not attempt to simulate the dynamic processes involved in overall cassava growth and development to determine yield. Root yield is generally determined at a fixed harvest time and is related to empirically established relationships between the environmental conditions during the growing season. These relationships may be complex as is the case of the QUEFTS based models that establish associations between nutrient uptake over the growth cycle and yield. We refrain from discussing the structure of these models in detail, as it varies substantially depending on the data used to specify and fit the models, but details of the static or regression-based models are provided in Sect. 3.

## 3 Cassava models

A total of 18 cassava models were identified in the literature review. This section describes briefly the main dynamics simulated by each model. A detailed summary of the processes and variables that are included in each cassava model is presented in Table 1. In the section below the model numbers correspond to the reference numbers shown in the first column of Table 1.

**Table 1:** Summary of cassava models and the processes that are simulated.

No.	Model	Reference	Environmental factors								Time step^3^	Management		Env^1^
			T^1^	P^1^	R^1^	VPD^1^	SWD^1^	Nutrients^2^	IC^1^	Pest		PD^1^	Cul^1^	
1	Cock	Cock (1978, 1979)	–	–	–	–	–	–	–	✓	W	✓	✓(3)	1
2	Fukai and Hammer	Fukai and Hammer (1987)	✓	✓	✓	–	✓	N	–	–	W	–	–	1 (14)
3	Gutierrez	Gutierrez et al. (1988b)	✓	–	✓	–	✓	N	–	✓	D	–	–	1
4	SUCROS	Gijzen et al. (1990)	–	–	✓	–	–	–	–	–	D	–	✓(4)	1
5	LINTUL	Ezui et al (2018); Adiele (2020)	✓	–	✓	–	✓	N, P, K	–	–	D	✓	–	1(2)
6	GUMCAS	Matthews and Hunt (1994)	✓	✓	✓	✓	✓	–	–	–	D	✓	✓(10)	2(4)
7	HyCAS	Matthews and Lawson (1997)	–	✓	✓	✓	✓	N	✓	–	D	✓	–	1
8	SIMANIHOT	Gabriel et al. (2014); Tironi et al. (2017a, b)	✓	✓	✓	–	✓	–	–	–	D	✓	✓(5)	2(4)
9	DSSAT-CROPSIM	Hoogenboom et al. (2019b, 2019a)	✓	✓	✓	✓	✓	N	–	–	D	✓	✓(16)	2
10	DSSAT-MANIHOT	Hoogenboom et al. (2019b, 2019a); Moreno-Cadena (2018); Moreno-Cadena et al. (2020)	✓	✓	✓	✓	✓	N	–	–	D	✓	✓(11)	3
11	Gray	Gray (2000, 2003)	✓	–	✓	✓	✓	–	–	–	DH	–	–	1
12	SIMCAS	Santhosh Mithra et al.(2013)	✓	✓	✓	–	✓	N, K	–	–	D	–	✓(3)	1
13	FAO Agroecological zone	Visses et al. (2018)	✓	✓	✓	–	✓	–	–	–	10	–	–	8(7)
14	DYNCAS	Connor (2019)	✓	–	✓	✓	✓	–	–	–	DH	–	✓(2)	1
15	Boerboom	Boerboom (1978)	–	–	–	–	–	–	–	–	S	–	✓(24)	1(7)
16	Manrique	Manrique (1992)	✓	–	✓	–	–	–	–	–	S	–	–	3
17	QUEFTS	Byju et al. (2012)	–	–	–	–	–	N, P, K	–	–	S	–	–	4(4)
18	Modified QUEFTS	Ezui et al. (2017)	–	–	–	–	–	N, P, K	–	–	S	✓	✓(2)	3(2)

No.	Model	Reference	Processes													
			Development					Growth								
			Indet. crop^1^	Lf^1^	Lf coh.^1^	Lf acce. sen.^1^, ^4^	Br^1^	Qual. PM^1^	Photo^1^, ^5^	Resp ^1^	Partitioning^6^	Root 1	Lf. size 1	DW 1	FW^1^	Sth 1
1	Cock	Cock, 1978; 1979)	✓	✓	✓	S	✓	–	–	–	S-O	–	✓	✓	–	–
2	Fukai and Hammer	Fukai and Hammer (1987)	–	✓	–	S, T, W	–	✓	RUE	–	LAI	–	✓	✓	–	–
3	Gutierrez	Gutierrez et al. (1988b)	✓	✓	✓	–	✓	–	DD	✓	Prt-age	✓	–	✓	–	–
4	SUCROS	Gijzen et al. (1990)	✓	–	–	S	–	–	CP	✓	Prt-age	–	✓	✓	–	–
5	LINTUL	Ezui et al (2018); Adiele (2020)	–	–	–	S, W	✓	✓	RUE	–	Prt-age, F-start	✓	✓	✓	–	–
6	GUMCAS	Matthews and Hunt (1994)	–	✓	✓	S	✓	–	RUE	–	S-O, F-start	✓	✓	✓	–	–
7	HyCAS	Matthews and Lawson (1997)	–	✓	✓	–	✓	–	RUE	–	S-O, F-start	–	✓	✓	–	–
8	SIMANIHOT	Gabriel et al. (2014); Tironi et al. (2017a), b	–	✓	✓	S, T	✓	–	RUE	–	S-O, F-start	–	✓	✓	–	–
9	DSSAT-CROPSIM	Hoogenboom et al. (2019b, 2019a)	–	✓	✓	S	✓	✓	RUE	✓	S-O, F-start	✓	✓	✓	–	–
10	DSSAT-MANIHOT	Hoogenboom et al. (2019b, 2019a); Moreno-Cadena (2018); Moreno-Cadena et al. (2020)	✓	✓	✓	S	✓	✓	RUE	✓	S-O	✓	✓	✓	–	–
11	Gray	Gray (2003, 2000)	✓	✓	–	–	✓	–	CP	✓	S-O, CE	✓	✓	✓	–	–
12	SIMCAS	Santhosh Mithra et al.(2013)	–	✓	–	S	✓	–	CP	–	S-O, F-start	✓	✓	✓	✓	–
13	FAO Agroecological zone	Visses et al. (2018)	–	–	–	–	–	–	RUE	✓	Fixed	–	–	✓	✓	–
14	DYNCAS	Connor (2019)	–	✓	–	–	✓	–	CP	✓	D-Ptr	–	✓	✓	–	–
15	Boerboom	Boerboom (1978)	✓	–	–	–	–	–	–	–	Fixed	–	–	✓	–	✓
16	Manrique	Manrique (1992)	✓	–	–	–	–	–	RUE	–	Prt-age based on R and T	–	–	✓	–	–
17	QUEFTS	Byju et al. (2012)	–	–	–	–	–	–	–	–	–	–	–	✓	–	–
18	Modified QUEFTS	Ezui et al. (2017)	–	–	–	–	–	–	–	–	–	–	–	✓	–	–

1 Simulated factors and processes include: T: Temperature, P: Photoperiod, R: Solar radiation, VPD: Vapor Pressure Deficit response, SWD: Soil Water Dynamics, IC: intercropping, PD: Plant density, Cul: Diverse cultivars (number), Env: Number of environments (the number in parenthesis is for model evaluation when the environments are different than for model calibration), Indet. crop: Indeterminate crop, Lf: Leaf development/appearance, Lf coh.: Leaf cohorts, Lf acce. sen.: Accelerated leaf senescence, Br: Branching, Qual. PM: Quality of the planting material, Photo: Photosynthesis, Resp: Respiration, Root: Fibrous root growth, Lf. Size: Leaf size, DW: Dry weight, FW: Fresh weight, Sth: Starch content, ✓: Yes, -: No.

2 N: Nitrogen, P: Phosphorus, K: Potassium.

3 S: Static, D: Daily, W: Weekly, DH: Daily with some variables estimated hourly, 10: 10 days.

4 Accelerated leaf senescence due to: S: Shading, T: low temperature, W: water stress.

5 RUE: Radiation Use Efficiency, DD: Demand-driven, CP: canopy photosynthesis.

6 S-O: Spill-over, LAI: Leaf Area Index, Prt-age: partitioning modified by plant age, R: solar radiation, T: temperature, F-start: fixed start of root filling, CE: Chanter’s growth equation, d-Ptr: dynamic partitioning.

### 3.1 Dynamic models

#### 3.1.1 Cock model

The model developed by Cock et al. (1979) (model 1) at CIAT was the first dynamic phenological model of cassava. This model was largely designed to provide breeders with guidelines on genetically controlled traits associated with cassava yield, and hence emphasizes both characteristics which are specific to cassava and those for which there is genetic variation. This model does not consider variation in soil and weather conditions and only one management option, i.e., plant spacing. Experimental data from fields close to the equator were used to set the model parameters. The development of the leaf area index (LAI) is simulated based on the rate of leaf formation per apex, number of apices per plant, the number of primary shoots per unit area of land, the area of the individual leaves, and longevity of the leaves. The individual area of a leaf increases up to four months after planting (MAP) and then decreases until harvest, while the leaf appearance rate decreases as a function of plant age. The time to each branch and the number of branches at each branching point are cultivar traits. All branches are equal with similar characteristics for the cohorts of leaves that form at the same time. The senescence rate is accelerated for shaded leaves that intercept less than 5 % of the incident radiation.

The model initiates crop growth with 1 g per plant of reserves from the planting piece. A standard curve derived from field experiments was used to estimate the overall crop growth rate from simulated LAI values on a weekly basis. This crop growth rate represents the assimilate production based on the radiation intercepted by the simulated leaf area. The biomass required to produce the new leaves and their supporting structure is estimated. If this estimated biomass is greater than the calculated maximum crop growth rate for the period, the leaf formation rate is reduced so that actual top growth rate is equal to estimated crop growth rate. If the crop growth rate is greater than the assimilate requirements for the top growth rate, the excess is allocated to storage root growth. This is commonly known as the spillover model, which contrasts with a dynamic allocation to the distinct organs (Evans, 1990).

The main outputs of the model are leaf number, branching time, number of apices, LAI, aboveground biomass, and storage root biomass. The model predicts the response to interplant competition with a decrease in the harvest index and consequently root yield under high planting densities, suggesting that the spill-over model for biomass partitioning is effective (Cock et al., 1979). The model was used to simulate various branching types. These simulations indicated that late branching phenotypes was optimal for yield. Field trials in which branching was manipulated by removing branches from a heavy branching type confirmed that later branching was associated with higher yields (Tan and Cock, 1979). Late branching phenotypes are now the standard cassava plant type that breeders aim for (Adiele et al., 2020; Phoncharoen et al., 2019). The model was also used to predict which type of pest damage would be most severe to define the focus of cassava pest and disease management programs (Cock, 1978).

#### 3.1.2 Fukai and Hammer

Fukai and Hammer (1987) (model 2) developed a dynamic model to predict yield for a wide range of environmental conditions in the tropics and subtropics. This was the first model to consider weather and soil variability. The model uses empirical relationships to establish the development of leaf area and accompanying stem growth. The biomass production is estimated from the relationship between crop growth rate and LAI modified by stress factors. The distribution of biomass is based on empirically determined partitioning factors.

The model inputs that describe the environmental conditions are planting date, temperature, solar radiation, daylength, rainfall, pan evaporation, bulk density, soil depth, permanent wilting point, field capacity, and initial soil moisture. The model does not distinguish between varieties and the input parameters are based on the two most widely grown varieties when the model was originally developed, i.e., MAUS7 and MAUS10. The plant parameters required are leaf longevity, specific leaf area, water stress index, potential crop growth rate, and assimilate partitioning factors. Initial growth is dependent on the reserves of the stick that is planted. A series of algorithms determine crop establishment as it relates to temperature, daylength, and the soil and plant water balance. Drought stress in this model is estimated as the ratio of maximum root water uptake to potential transpiration; the maximum water uptake is defined by the available soil water. The model introduces the concept of a heat sum with no growth below an average temperature of 10 °C. Biomass production is estimated from the crop growth rate as a function of LAI with a maximum crop growth rate of 20.6-21.7 g m^−2^ d under optimum conditions. Fukai and Hammer (1987) note the similarity of their model to the Cock model under optimal conditions. The crop growth rate is modified by water stress, solar radiation, and temperature. The partitioning of biomass uses empirical relationships, which makes it difficult to apply the model to cultivars or regions that are different from the ones that were used to develop the equations. Partitioning to the storage roots is reduced under water stress and increases with nutrient restrictions and low temperatures because of an expected physiological decrease in shoot growth. A response to photoperiod is included in the model, with long days associated with an increase in top growth and a reduced allocation to the storage roots. Leaf life in the model is reduced by shading at high LAIs, water stress and temperatures below 15 °C. As leaves senesce, 30 % of the assimilates are re-translocated and are available for growth of the stems and storage roots. The model predicted cassava yield under varying conditions in Northern Australia well.

#### 3.1.3 Gutierrez

The dynamic and phenological model developed by Gutierrez et al. (1988b) (model 3) was designed to describe the development and growth of cassava, as affected by weather, soil water and nitrogen. The Gutierrez et al. (1988b) model evaluates damage and control of exotic cassava pests and uses population theory as a framework that integrates plant physiology into a population dynamics model. They used the concept of population ecology to represent cohorts of cassava leaves with different ages as a population with changing birth and death rates. New leaves are produced over time with the number of leaves for each cohort related to the branching pattern. The leaf area is determined from the number of leaves (birth rate), their expansion (mass), and senescence and abscission (death rate in this model). The overall phenology is through branching, which defines the potential leaf production rate and architecture of the plant. First branching is predicted when the available carbohydrates (photosynthesis plus reserves) are three times greater than the demand, while additional branching is based on thermal time. We note that the factor of three for the excess carbohydrate over demand appears large and suspect that the demand is leaf demand, as witnessed by the earlier statement that a key feature of the models is that total demand is related to leaf demand (Gutierrez et al., 1988b).The potential demand for assimilate and nitrogen is a function of the leaf production rate. However, the leaf appearance rate and the growth demands can be reduced because of a diminished production of assimilates due to weather or nutrients restrictions, competition with other plant organs and pest damage. The model has a fixed distribution of biomass that is based on growth stage. The growth of stems, feeder roots, and storage roots are proportional to the leaf demand. The potential demand for assimilate is compared to the total assimilates and reserves that are available for growth to determine the overall growth of the plant. The increase in leaf area and the actual growth of all organs is reduced proportionally if the assimilate is less than the demand. If the demand is less than the supply, the reserves are increased. Thus, the Gutierrez model is the first to simulate a reserve carbohydrate pool.

Water and nitrogen (N) uptake are simulated as a predation process using a metabolic pool to define the assimilation. This model was the first that incorporated the idea that nutrient restrictions mainly affect LAI development with N stress manifested as a reduction in growth demand. For this model it provided better results compared to a reduction in photosynthesis used in other plant models (Gutierrez et al., 1988b). A water balance model is included in the model to define water stress which influences canopy development and carbon assimilation. The net result of the new leaf area that is formed and the leaves that are abscised, results in the LAI that is used to estimate carbon assimilation based on light interception and photosynthesis. This model includes respiration and the metabolic costs of biomass production.

The Gutierrez et al. (1988b) crop model was linked to population dynamics models of two cassava pests using the concept of trophic levels (Gutierrez et al., 1988a, 1988c) and provided insights into the implementation of the immensely successful program to control the cassava mealy bug in Africa (Gutierrez et al., 1994).

#### 3.1.4 Wageningen ‘school of C. T. de Wit’ models

##### 3.1.4.1 SUCROS

Gijzen et al. (1990) modified the SUCROS model (Simple and Universal CROp growth Simulation model) (Penning de Vries and Laar, 1982) for cassava (model 4). The dynamic, empirical, SUCROS cassava model is based on the experimental data from Veltkamp (1986) for one location (CIAT, Palmira, Colombia), which may reduce its application to other environments. The model simulates plant growth as a function of gross photosynthesis, which is the main driver of the SUCROS model. In contrast to most other cassava models, it also simulates maintenance and growth respiration. The products of photosynthesis, after accounting for respiratory losses and conversion to biomass of distinct composition in the various organs, are distributed to the plant organs using empirically determined partitioning coefficients dependent on development stages of before and after commencement of tuber filling. From the accumulation of biomass in the leaves, the leaf area is determined which then provides the basis for the next round of photosynthesis. The SUCROS model also considers the translocation of assimilates from senescing leaves to other plant components. The inputs of the model include solar radiation, latitude, and day of the year. The crop parameters in the model are dry matter partitioning table, assimilate conversion, respiration rate per plant organ, rate of photosynthesis at light saturation, light use efficiency, extinction coefficient and initial start of starch accumulation. The model generates outputs of dry weight for storage roots, leaves, stems, and fibrous roots. A sensitivity analysis of the model showed that the parameters for radiation use efficiency, rate of photosynthesis at light saturation, and the extinction coefficient are the most relevant for dry matter production.

##### 3.1.4.2 LINTUL

The LINTUL cassava model was developed to enhance the knowledge of cassava growth and yield considering drought stress and different planting dates under rainfed conditions in West Africa and to ultimately improve commercial cassava production (Ezui et al., 2018). The original LINTUL model (Light INTerception and UtiLization model) is based on the SUCROS model. However, rather than simulating detailed photosynthesis and respiration processes, the model uses the concept of radiation use efficiency (Spitters and Schapendonk, 1990). LINTUL assumes a linear relation between the intercepted light and the crop growth rate, where the intercepted solar radiation is based on LAI and the extinction coefficient. Ezui et al. (2018) modified the version of LINTUL developed for potatoes (Spitters and Schapendonk, 1990) to include the effect of drought stress on cassava. This dynamic empirical model was released as LINTUL-2 (model 5).

The development of the leaf canopy during the initial growth period after sprouting is an empirically established exponential increase in LAI modified by the thermal time. After the initial growth period, leaf area growth depends on the available assimilates for leaf growth. Biomass production is determined by the standard LINTUL photosynthesis and respiration procedures. The distribution of biomass to the individual plant organs is based on partitioning factors that are modified as a function of the development stage. The development stages are simulated as a function of thermal time and include the phases from planting to sprouting, sprouting to first branching, and first branching to maturity. At the juvenile stage, the biomass partitioning to leaves, stems and fibrous roots is prioritized. A fixed root initiation parameter expressed in thermal time is used and after root initiation, which generally occurs around four months after planting for the conditions for which the model was developed, the priority for biomass partitioning is modified to favor tubers. LAI is estimated from biomass production and partitioning and is used to determine the radiation interception and biomass production for the next period.

The LINTUL-2 model requires daily total rainfall, solar radiation, temperature, initial soil water content at planting, and initial planting stick weight as input. The model has 27 crop parameters related to leaf growth, biomass partitioning and cardinal temperatures. In addition, eight soil parameters that define the drainage rate, a drought stress factor, and the soil water contents for air dry conditions, wilting point, and field capacity, are necessary to run the model. The LINTUL model for cassava assumes a determinate growth of the crop with a default maximum age of 4320 thermal units or 360 days under optimal conditions which are defined with an optimum temperature of 27 °C and a base temperature of 15 °C. Under drought stress, biomass production is decreased through the application of a transpiration reduction factor, which is the ratio of the actual over the potential transpiration. Furthermore, under drought conditions there is a higher priority to the allocation of biomass to fibrous root growth to be able to explore a larger volume of soil for water, thereby reducing the proportion of carbohydrates allocated to the shoots and storage roots. During prolonged dry periods, the model assumes a dormancy period. For the recovery from dormancy when there is an increase in available soil water, LINTUL simulates the translocation of assimilates from roots to support shoot growth and the development of new leaves.

The main outputs of the model are LAI, total and storage root biomass, cumulative soil evaporation and cumulative soil transpiration. An initial evaluation of the LINTUL-2 model under drought conditions for two locations in Togo showed a reasonable fit with a normalized root mean square error (NRMSE) of 0.134 for the storage roots which represents 13 % of the measured storage dry weight (Ezui et al., 2018).

Adiele et al. (2020; 2021) developed an updated version of LINTUL-2, which includes the effect of nitrogen (N), phosphorus (P) and potassium (K) on the growth of cassava. This new model simulates accurately the storage root yield with a root mean square error of prediction (RMSEP) of 3.552 t dry matter roots ha^−1^ for three locations in Nigeria.

#### 3.1.5 GUMCAS

##### 3.1.5.1 Original GUMCAS

GUMCAS (from *gumaya* in Tagalog, which means simulate) (model 6) (Matthews and Hunt, 1994) is a dynamic, phenological model. GUMCAS builds on the Cock model but includes weather and soil variables to better understand the response of the crop to distinct environments.

Phenology is simulated as development days that depend on the heat sum above a threshold temperature of 13 °C. The model uses the concept of distinct clocks for three growth phases: planting to emergence; emergence to first branching; and first branching to harvest maturity. The branching rate is assumed to be constant after the first branching level with time to first branching modified by temperature and photoperiod. The maximum leaf size is reached at 70–80 after emergence, while the minimum leaf size is attained at 300 days after emergence. The leaf size is based on a generalized empirical relationship with the heat sum modified by the available assimilate compared to an empirical algorithm that is used in the Cock model. Leaf size is known to increase markedly on recovery from a drought stress (Connor and Cock, 1981) and this is simulated with a compensatory factor for leaf size when a drought stress is alleviated. This provides an improved fit with experimental data when simulating the increase in leaf size at the beginning of the rainy season (Matthews and Hunt, 1994). Potential leaf longevity is a cultivar characteristic that is modified by shading and temperature. The model also simulates the effect of photoperiod with a reduction in the accumulation of thermal time during short days, which results in a reduction in leaf appearance and branching rates.

The simulated phenology determines the formation of new leaf area. New leaf area development is restricted by the availability of assimilates. LAI is estimated based on the new leaf area that is formed and the senescence and loss of older leaves. The crop growth rate follows the Fukai and Hammer model as a function of LAI, temperature, solar radiation, and drought stress. The drought stress factor is estimated by comparing the potential water uptake rate with the potential transpiration rate using the Ritchie water balance approach (Ritchie, 1998). The GUMCAS model was the first cassava model that considered the effect of VPD on stomatal conductance, using a daily average VPD effect as a multiplier on crop growth.

The spillover model used in the Cock model describes the distribution of biomass. Stem growth is defined as a fraction of leaf growth, which in turn is a function of crop age based on thermal time. The model has 23 crop parameters related to photoperiod sensitivity, developmental time to emergence and branching, leaf appearance rate, number of branches, VPD sensitivity, crop growth rate, leaf size, leaf duration, partitioning of biomass to the stems and fibrous root growth.

##### 3.1.5.2 HyCAS

The HyCAS model (model 7) is an intercropping model based on the GUMCAS model for cassava and the Hybrid tree model (Matthews and Lawson, 1997). HyCAS considers the competition between crops and trees for N, water, and light. The radiation that reaches the cassava crop is estimated after subtracting the intercepted radiation of the trees from the total incident solar radiation. Water uptake and N for the trees and cassava are based on the size of the root system assuming that the root inflow rate is the same for both species. The main outputs of the model are total amount of water uptake during the growing season, total biomass of trees and crop, and nitrogen uptake. Until now it is the only intercropping model for cassava as part of an agroforestry system. Matthews and Lawson (1997) generated different scenarios with various tree densities, fertilizer applications and tree ages. However, they did not provide any information with respect to model performance or a more detailed description of the model.

##### 3.1.5.3 SIMANIHOT

SIMANIHOT (model 8) was developed based on the original GUMCAS model to compare it with and to incorporate some of its functions into the DSSAT model that was available at that time (Gabriel et al., 2014). The developers evaluated both models for subtropical conditions of Southern Brazil and incorporated the specific traits of the local varieties. This phenological model developed in the south of Brazil is the first cassava model that incorporates the effects of a cool winter period. The model uses three development “clocks” to define leaf appearance, branching time and the initiation of storage roots. The model also includes new equations for the simulation of leaf appearance and leaf senescence to improve the simulation of those processes under subtropical conditions. The leaf appearance rate per apex was modified to include a nonlinear response to temperature and a cultivar parameter for maximum leaf appearance rate. Leaf senescence was modified by tracking the age of each cohort of leaves independently and with the rate of senescence hastened at cooler temperatures below 5 °C. The original SIMANIHOT model did not consider water or nutrient restrictions and did not include the original VPD function from the GUMCAS model due to the generally humid conditions of Rio Grande, Brazil. Initial evaluation of the model for southern Brazil showed a good performance with a root mean square error (RMSE) of 2 t ha^−1^ for the storage roots dry weight.

Tironi et al. (2017a) modified the original SIMANIHOT model (Gabriel et al., 2014) and added two soil water balance methods including the Thornthwaite and Mather (1955) approach, and Ritchie (1998) model. The model has 26 crop parameters that include the maximum water uptake by the roots, developmental time to emergence, branching, and beginning of starch accumulation, number of branches, specific leaf area, leaf size, maximum crop growth rate, leaf duration, senescence sensitivity, leaf appearance rate, and stem/shoot ratio. The soil parameters for the Tironi et al. (2017a) model are the soil water content at 650 kPa and at field capacity. The leaf appearance rate, leaf area, leaf size and crop growth rate are reduced when a minimum threshold value of the fraction of available soil water is reached based on the soil water balance methods. The main outputs of the model are number of leaves, number of apices, LAI, and biomass of leaves, stems and roots. The model performed well when simulating yield for five cultivars grown in two locations in southern Brazil. The two new soil water balance modules also showed a good prediction for soil water content.

The SIMANIHOT model incorporates response to elevated CO_2_ levels and has been used to evaluate the impact of climate change on cassava production in the state of Rio Grande do Sul in Brazil (Tironi et al., 2017b). Largely due to the increase in temperature, yield was predicted to increase under future climate scenarios.

##### 3.1.5.4 DSSAT-CROPSIM

The Cropping System Model (CSM)-CROPSIM (model 9) of the Decision Support System for Agrotechnology Transfer (DSSAT) uses the CROPSIM model template (Hunt and Pararajasingham, 1995) implemented as a module in CSM (Jones et al., 2003), the main engine of DSSAT (Hoogenboom et al., 2019a, 2019b; Jones et al., 1998). The dynamic, phenological, CROPSIM module was modified for cassava based on the GUMCAS model (Matthews and Hunt, 1994). However, the CROPSIM module differs substantially from the GUMCAS model: the use of air instead of soil temperature for estimating germination and emergence; the developmental rate is not modified under drought stress; an equation to define potential leaf size was added; new equations that define the effect of photoperiod and fibrous root growth; decreased concentration of leaf nitrogen content as the plant ages; and distinct treatment of crop growth phases or stages. In addition, the CSM-CROPSIM-Cassava model has a parameter to define the initiation of the storage root, which does not correspond to the spill-over strategy of GUMCAS. The model has distinct cardinal temperatures for germination, development, leaf growth and photosynthesis, while GUMCAS has only one set of cardinal temperatures. The leaf appearance rate is reduced as the plant ages in both models, but the equations are different. In GUMCAS, a new leaf reaches its final size on the same day that it appears while in CSM-CROPSIM-Cassava they expand over time until they reach full expansion. The potential leaf size curve in GUMCAS uses chronological time, while CSM-CROPSIM-Cassava estimates the potential leaf size based on thermal time. In GUMCAS, the stem growth is defined as a fraction of the leaf growth modified by the crop age while CSM-CROPSIM-Cassava the fraction is a constant for each cultivar.

Both GUMCAS and CSM-CROPSIM-Cassava consider the photoperiod effect. However, GUMCAS uses a linear reduction in the accumulation of thermal time during short days while CSM-CROPSIM-Cassava uses an exponential equation. Similar to GUMCAS, the CSM-CROPSIM-Cassava also simulates drought stress as a function of potential transpiration and potential root water uptake based on the Ritchie (1998) approach. CSM-CROPSIM-Cassava has 85 species parameters related to cardinal temperatures, photoperiod effect, fibrous root growth, leaf senescence, extinction coefficient, reserves distribution, photosynthesis, and water and nitrogen stress indexes. In addition, 10 ecotype parameters define the canopy height, standard nitrogen concentration in storage roots, and number of apices. Finally, the model has 21 cultivar parameters that specify photoperiod sensitivity, branching times, partitioning fraction to the storage roots, leaf size, leaf appearance rate, leaf petiole fraction and leaf duration. The main outputs of the model are LAI, number of storage roots, leaves, stems, fibrous and storage root biomass.

##### 3.1.5.5 DSSAT-MANIHOT

The CSM-MANIHOT model (model 10) (Hoogenboom et al., 2019b, 2019a; Moreno-Cadena, 2018; Moreno-Cadena et al., 2020) is also part of the DSSAT Crop Modeling Ecosystem and is based on the CSM-CROPSIM-Cassava model, using the same environmental inputs variables, i.e., daily total solar radiation, minimum and maximum air temperature, and daily total rainfall. However, CSM-MANIHOT-Cassava model represents a further advancement of the CSM-CROPSIM-Cassava model as it simulates specific growth and development responses that are unique characteristics of cassava. The model simulates basic phenology of cassava based on the Cock model (Cock et al., 1979). Each node is a member of a cohort; nodes that are formed on the same day are considered as one cohort and the nodes for one cohort are all equal in size during development. The leaf appearance rate is based on a saturation growth rate function that represents the indeterminate growth of cassava with a reduction in the node appearance rate when the crop ages. This function was created based on the data reported by Irikura et al. (1979) for four varieties grown under different temperatures using the daily accumulated thermal time and thermal age of the plant, which decreases the leaf appearance rate when the crop ages. This function also includes a cultivar parameter that allows for the differentiation of varieties that have different leaf development rates (Moreno-Cadena, 2018). Once the third branch level has been initiated, the branching rate (in thermal time) is assumed to be constant. The CSM-MANIHOT-Cassava model uses diverse cardinal temperatures for leaf age, leaf growth and branching. The node growth rate is defined as a logistic function with a modified rate based on the leaf number when the node appears and the age of the node. The maximum leaf size is reached at 900 degree days using a base temperature of 13 °C and an optimum temperature of 24 °C and is reduced as the crop ages (Moreno-Cadena et al., 2020).

The CSM-MANIHOT-Cassava model considers the radiation use efficiency concept to estimate the daily production of assimilates. The model includes a spill-over strategy that was originally defined in the Cock model (Cock et al., 1979) with no parameter to define the initiation of the storage roots. New growth of leaves, stems and fibrous roots has priority over the growth of storage roots. If the daily assimilates do not satisfy the demand for leaf, stem and fibrous growth, the actual growth of those organs is proportionally reduced. When the demand is less than the assimilates that are produced, the additional carbohydrates are relocated for storage root growth (Moreno-Cadena et al., 2020). The demand for leaf growth is defined by the potential leaf area increase in all the cohorts divided by the specific leaf area. The potential stem growth is the sum of the potential node growth rate for all the cohorts. The demand for fibrous root growth is defined as 10 % of the demand for aboveground growth for leaves and stems (Moreno-Cadena et al., 2020).

Drought stress is based on the available soil water content instead of the ratio of actual and potential transpiration that is used in the other CSM models of DSSAT, the Fukai and Hammer model, and the LINTUL model. This stress factor delays germination and branching, slows leaf appearance rate, and it can also reduce leaf size and photosynthesis (Moreno-Cadena et al., 2020). The CSM-MANIHOT-Cassava model also includes nitrogen restrictions, but they are based on the original CSM-CROPSIM model and requires further improvement and evaluation.

The current released version of the CSM-MANIHOT-Cassava model requires 77 species parameters to define water and nitrogen stress thresholds, leaf, and root senescence, VPD effect, and root water uptake. In addition, eight ecotype parameters specify the radiation use efficiency, base temperature for leaf development, extinction coefficient, thermal time to germination, and the sensitivity to photoperiod. Finally, 14 cultivar parameters define the thermal time to branching, the number of branches per branching point, maximum individual leaf size, specific leaf area, leaf duration, leaf appearance rate, leaf petiole ratio, individual node weight and length. The main outputs of the CSM-MANIHOT-Cassava model are the same as for the CSM-CROPSIM-Cassava model.

#### 3.1.6 Gray

Gray (2000) (model 11) developed and compared two dynamic, phenological, models that differ in their allocation of carbohydrates to the storage roots: one is based on the spill-over strategy and the second one uses the Chanter’s equation (Chanter, 1976) to estimate the partitioning of biomass to the roots. Gray (2000) hypothesized that the spill-over strategy cannot account for the effect of storage root sink strength and, therefore, he proposed the Chanter’s equation, which was originally developed for mushrooms. In this model the growth of the storage root is dependent on both the carbohydrate reserve pool and the efficiency of storage root production (ESPR). ESPR is similar to the partitioning of the biomass constant that was used by Boerbooom (1978), with storage root growth modified based on the storage root structural mass, and a second factor that “depends on the passage of time and can be interpreted as the progression or state of storage organ differentiation and development” (Gray, 2000). This approach assumes that allocation depends on the two processes of substrate transport and chemical conversion.

Similar to the Cock model (Cock et al., 1979), the leaf growth rate in both models is based on the rate of leaf formation, leaf longevity, leaf size and number of apices. However, the Gray models incorporate the concept of thermal time instead of chronological time to define the leaf appearance rate. Both models have the same node development with the leaf formation using a modified strategy of Matthews and Hunt (1994). The potential leaf size is defined based on the leaf position, while the carbohydrates demand of the stems is estimated by the diameter and length of the nodes. Neither of the two models consider drought or nutrient stresses.

The environmental inputs for both models are the daily mean temperature and solar radiation while the main outputs are LAI, and biomass of leaves, petioles, stems, fibrous and storage roots. The spill-over model has a total of 47 parameters, while the Chanter’s model has 52. However, the spill-over model just considers eight crop parameters for calibration including carbon concentration when the light saturated rate of photosynthesis is reduced to 50 %, the number of apices and primary stems, internodal length of the stems and branches, inhibition factor of the photosynthesis based on the substrate concentration, and maximum leaf senescence rate. The Chanter’s model has three additional parameters for calibration in comparison to the spill-over with two constants for storage root growth and one of substrate concentration in the storage roots. A sensitivity analysis of the variability in the simulated total biomass and yield due to the adjustment of ±5 % in the values obtained for all the input parameters showed that the model based on the Chanter equation is more sensitive to the parameters that affect storage root growth than to the ones that define the assimilate demand (e.g., concentration of assimilates for maximum leaf growth, internodal lengths for stems). In contrast, the spill-over model is more sensitive to parameters that are related to the photosynthesis rate and assimilate demand in comparison to storage root growth.

Gray (2003) added the simulation of the water balance to the original models (Gray, 2000). The evapotranspiration is estimated using the Campbell equation (Campbell and Norman, 1998). The water balance model modifies the stomatal conductance according to the daily average vapor pressure deficit (VPD) and the soil water potential. The modification of stomatal conductance directly influences both photosynthesis and evapotranspiration. Furthermore, the leaf appearance rate is reduced with an increase in vapor pressure deficit.

#### 3.1.7 SIMCAS

The dynamic, phenological, SIMCAS model (model 12) for cassava was developed by Santhosh Mithra et al. (2013) to specifically addresses the weaknesses of previously developed cassava models. The SIMCAS model emphasizes the critical balance between the sink capacity and source potential of cassava and how this is modified by field conditions. Furthermore, SIMCAS recognizes the limitations of empirical models that require recalculation of parameters when evaluating a new variety or environment, while it builds on the experiences with previous models, especially the GUMCAS model.

The simulation of phenology is based on thermal time. The effect of photoperiod follows the approach of Matthews and Hunt (1994) with a multiplicative factor for thermal time. LAI is a function of leaf appearance rate, leaf longevity, the number of apices, and leaf size. Shading reduces the LAI through an increase of leaf senescence and abscission. The leaf appearance rate is based on a cultivar parameter that defines the number of leaves at emergence. The optimum leaf appearance rate is defined at 28 °C, while any temperature either above or below 28 °C reduces the leaf appearance rate. Santhosh-Mitra et al. (2013) set the maximum leaf size at a given plant age, but the model does not modify the maximum leaf size through the growth period. The shading effect is based on the phyllotaxy of cassava. This effect does not consider the phototropism of cassava leaves, which can modify the leaf shading. The model modifies the number of branches according to the availability of carbohydrates; if the availability is greater than the demand, three branches are formed while if the availability is less than the demand, only two branches are formed.

Storage root initiation is simulated using the Boerboom (1978) approach based on a threshold plant weight. Once tuber growth is initiated, the spillover model is implemented with the aboveground organs as the main sink while stem growth terminates at a defined crop age. The model also simulates stresses due to drought, and N and K deficiencies. The drought stress is estimated based on the actual and potential evapotranspiration, while the N and K stresses are defined as the ratio between nutrient uptake and demand. The growth is limited by the available assimilate and the stress factors for N, K, and water. The inputs of the model are air temperature, sunshine hours, relative humidity, rainfall, total nitrogen, and potassium available in the soil, crop residue and fertilizers. SIMCAS considers 14 crop parameters including the number of leaves at emergence, the stem growth rate, thermal age for storage root initiation, maximum dry weight of fibrous roots, dry weight of fibrous root at emergence, the total number of roots, specific leaf area, maximum biomass of the stems, maximum individual leaf size, leaf duration, the number of nodes needed for branching, the number of shoots, and initial plant weight. Outputs of the model include LAI, branching times, and weight of leaves, stems, and storage roots.

The original model was evaluated for three cultivars in India that were grown between 1991 and 2002. The model showed a reasonable prediction for yield with a mean absolute percentage deviation between 13.2 % to 17 %.

#### 3.1.8 FAO agroecological zone

Potential and attainable yield for the main cassava growing areas of Brazil were estimated by Visses et al. (2018) using an adapted version of the FAO Agroecological Zone crop simulation model (model 13) (Doorenbos and Kassam, 1979). This is a dynamic, empirical model. LAI is based on empirical values for five growth stages including planting to beginning of root growth, aboveground growth, root thickening, physiological repose, and new vegetative growth. Thus, this model considers the hiatus during the cooler winter period or during a severe drought stress. Biomass production is determined from photosynthesis estimates at distinct LAI values that are modified by drought stress, temperature, and solar radiation. The main environmental inputs for this model are solar radiation, sunshine hours, mean air temperature, wind speed and relative humidity. This model is based on a 10-day time step although gross photosynthesis for potential yield is simulated daily based on the ratio of total number of sunshine hours and photoperiod, extraterrestrial solar radiation, and photosynthetic efficiency. Gross photosynthesis is modified based on LAI and maintenance respiration. The maintenance respiration uses 40 % of the gross photosynthesis when the temperature is below 20 °C and increase to 50 % for higher temperatures.

This model assumes a fixed harvest index of 60 % with a dry matter content of 40 % in the roots. The attainable yield is simulated by multiplying potential yield with a water deficit factor, which is modified for each phenological stage as a function of the ratio of actual and potential evapotranspiration. The evaluation of the FAO Agroecological Zone model with data from 41 growing seasons for seven locations of Brazil resulted in a mean error of 2.1 t ha^−1^.

#### 3.1.9 DYNCAS

Connor (2019) recently developed a new cassava model, called DYNCAS 1.0 (model 14), which simulates phenology, growth and a soil and plant water balance. The model uses hourly weather variables as input, except for rainfall, which is based on a daily total. The DYNCAS model, unlike the other cassava models, requires daily pan evaporation and VPD. The model has 40 parameters that define the phenology and architecture of the plant, the production and distribution of assimilates, and the plant and soil water balance. Photosynthesis is based on a single leaf response that includes an exponential reduction in the intercepted radiation for the lower layers of the canopy. Plant density is fixed at 10,000 plants ha^−1^. Phenology, leaf appearance and branching rates are simulated based on thermal time; the leaf appearance rate is decreased as the crop ages and the branching rate is reduced under drought stress. The model considers a maximum of five branching levels and after the fifth branch has formed the simulation of new leaf appearance is terminated, thus representing cassava as a determinate crop. Rooting depth increases at a constant rate until the maximum soil depth is reached. Root growth and senescence are simulated as a function of thermal time using the soil temperature.

A stomatal response to both leaf water potential and VPD is included in the model which modifies both photosynthesis and transpiration. The partitioning of assimilates is based on two parameters for each organ: one defines the maximum uptake while the other is the assimilate concentration at half of the maximum uptake rate. A proportion of the carbohydrates in senescing leaves and roots is mobilized to the reserves. However, remobilization of assimilates from other organs is not considered in the model because of insufficient information about this process in the literature. The model simulated drought conditions with reasonable predictions for soil water content, and biomass of leaves, stems, and storage roots for one cultivar grown in Colombia (R^2^ of 0.94 for the biomass components).

### 3.2 Static or regression-based models

#### 3.2.1 Boerboom

The cassava model developed by Boerboom (1978) (model 15) is one of the earliest published models being developed at the same time as the Cock model. The model defines a short initial period with no tuber growth followed by a prolonged period of tuberous root growth. The initiation of tuberous root growth is defined by a threshold total plant weight above which tuberous root growth begins. After initiation, tuberous root growth is a constant fraction of new biomass; this constant is considered as the efficiency of root production (ESRP).

#### 3.2.2 Manrique

Manrique (1992) developed a statistical model based on a partial linear regression between the production and partitioning of biomass and temperature and solar radiation (model 16). The model is based on experimental studies that were conducted at three different elevations in Hawaii. The model is static with simulated LAI and biomass based on different sets of equations for 60-day periods. Although the equations include individual coefficients for the response to temperature and solar radiation, they do not consider the interactions between these correlated input factors. In addition, the model does not explain the reduction in LAI after reaching a maximum value around 180 days after planting. This reduction in LAI can be the result of a decrease in the leaf appearance rate and an increase in leaf senescence, which are processes that are not simulated by this model.

#### 3.2.3 QUEFTS models

##### 3.2.3.1 QUEFTS

The static model for Quantitative Evaluation of the Fertility of Tropical Soils (QUEFTS) originally developed for maize (model 17) (Janssen et al., 1990) was adapted for cassava by Byju et al. (2012). The latter was evaluated for different regions of India and provided a reasonable improvement of NPK fertilizer recommendations for cassava.

The QUEFTS model is based on the relationship between nutrient uptake and yield. QUEFTS estimates crop yields as a function of the supplies from the soil and fertilizers of N, P and K, the internal nutrient use efficiencies (yield to uptake ratio), and the attainable yields based on the location and its climate. The model follows fours steps to determine crop yield. In the first step, QUEFTS estimates the potential supply of each nutrient (N, P and K) from soil and fertilizers. In the second step, the potential supply of a given nutrient is used to calculate the uptake of that nutrient by considering the interactions with the two other nutrients (e.g., uptake of N is defined as limited by the pair of nutrients P and K; uptake of P by N and K; and uptake of K by N and P). In the third step, QUEFTS calculates two yield values for each nutrient uptake: one for the maximum accumulation of the nutrient in the plant (also called internal efficiency for maximum accumulation), and the other one for the maximum dilution of the nutrient in the plant (also called internal efficiency for maximum dilution). In the fourth step, the model calculates yield for pairs of nutrients following the yield ranges estimated in the third step, and the final yield is calculated as average yield of all pairs of nutrients. The soil supply of each nutrient for a specific site is determined from nutrient omission trials in which one nutrient is omitted while the other nutrients are supplied at a luxury level. The model assumes that when a nutrient is limiting in the soil, its internal use efficiency by the plant is maximized. Hence, in the case of the nutrient omission trials, the soil supply of the omitted nutrient is assumed to be equal to the total uptake by the plant of that nutrient when the other two are maximally supplied. The internal nutrient efficiency is extremely sensitive to the harvest index which is determined empirically as a constant for each variety.

##### 3.2.3.2 Modified QUEFTS

Ezui et al. (2017) (model 18) modified the QUEFTS model presented by Byju et al. (2012) and Janssen et al. (1990). They determined new values for the parameters for the internal efficiencies for maximum accumulation and maximum dilution of the nutrients based on the harvest index, and new equations for the supply of soil available nutrients. They first tested the equations from Janssen et al. (1990) for estimating soil supplies of N, P and K, but all nutrients in the soil were underestimated in comparison to measured values for experiments that were conducted in Togo, West Africa. They also tested the equations of QUEFTS developed by Byju et al. (2012) for estimating soil nutrient supply which also resulted in underestimating the N and P available in the soil while the K was overestimated. Then, Ezui et al. (2017) graphically estimated the soil supply of available nutrients and the maximum recovery fraction of the nutrients, respectively, as the intercept and the slope of the linear regression obtained by plotting the maximum nutrient uptake against the amount of nutrient applied in the training dataset. The modified QUEFTS (Ezui et al., 2017) showed a good fit between measured and simulated yield in the testing dataset under different fertilizer levels for two of the three zones evaluated in West Africa.

## 4 Model comparison

**Fig. 1:**
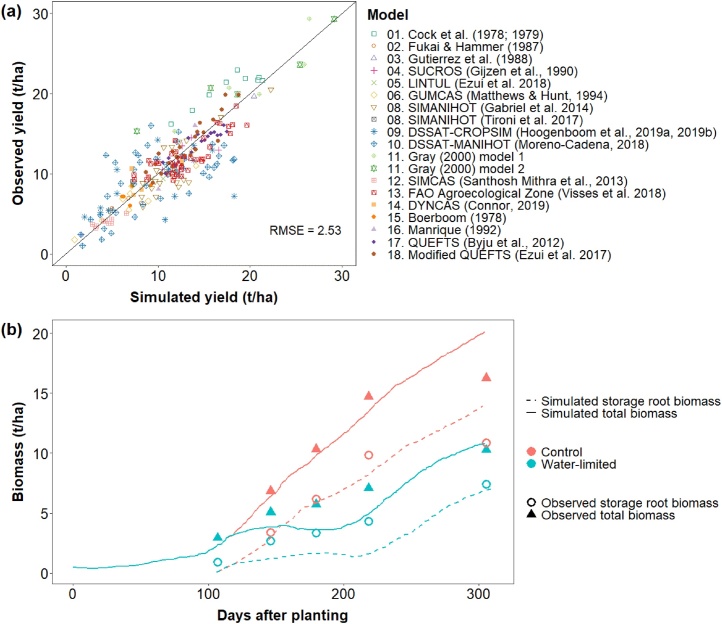
Simulated versus measured yield (t dry matter (DM)/ha) for 18 cassava models **(a)**; Note: The HyCAS model (#7) did not supply testing data results. Simulated (continuous lines) and measured (triangles) total biomass with the simulated (dashed lines) and measured (circles) storage root biomass of the GUMCAS model (#6) for an experiment with water stress **(b)** (reproduced from Matthews and Hunt (1994)).

The original calibration for all models were compared based on both the published simulated and measured dry yield for cassava (Fig. 1a). Because most of the models are not available, we were unable to run these models with the same data set for comparison of model performance. All models had a good fit with the measured data (RMSE = 2.53 t ha^−1^). The Cock et al. (1979) model tended to slightly underestimate yield and the CSM-CROPSIM-Cassava and CSM-MANIHOT-Cassava models showed considerable variability in the final cassava yield. Eight models were developed based on experimental data from only a single region with a relatively constant temperature and few crop cycles (models: 1, 3, 4, 5, 7, 11, 12, and 14; see Table 1 to identify the model number); four models consider the quality of the planting material for the initial simulation of growth and development (2, 5, 9, 10).

Some models define a time limit for the duration of the simulations; Fukai and Hammer (1987) restricts the simulations to 52 weeks (1 year) which is similar to the maximum duration simulated by DYNCAS (Connor, 2019). LINTUL has a maximum time of simulation of 4320 degree days (optimum temperature: 27 °C; base temperature: 15 °C) (Ezui et al., 2018), while the FAO Ageocological Zone method has the option to simulate a growing season between 12–18 months. Other models assume that cassava is a determinate crop; for instance GUMCAS set a phase where no more leaves appear in addition to a maturity stage with no more aboveground growth. QUEFTS define potential yield of cassava for a specific age of the crop. Santhosh Mithra et al. (2013) reduce the fibrous root growth when the storage roots initiate growth and terminate stem growth at a specific crop age. It is unclear based on the literature if the maturity stage of the SIMANIHOT model (Gabriel et al., 2014; Tironi et al., 2017a) was modified from the original GUMCAS model.

Models that consider the temperature and photoperiod effect on development are listed in Table 1. Two of the models that simulate the occurrence of branching modify the number of branches based on carbohydrate availability (3, 12). Seven models use the concept of leaf cohorts (1, 3, 6–10); twelve models (1, 2, 4, 5, 6–10, 12, 14) simulate the dynamics of leaf area, while nine models modify the potential leaf size during the growing season (1, 6, 7–11, 12, 14). Nine models include accelerated leaf senescense due to shading of the older and lower leaves in the canopy (1, 2, 4, 5, 6, 8–10, 12). In addition, two models (2, 8) increase the leaf senescence rate due to low winter temperatures, while two models (2, 5) increase the leaf senescence rate due to drought stress.

Seven models simulate the growth of fibrous roots (3, 5, 6, 9, 10–12). Gutierrez et al. (1988b) defined the proportion of root exploration in the soil as the percentage of light intercepted by leaves. LINTUL estimates the volume of water available in the soil based on rooting depth with increased root growth under water stress; however, the root volume is not estimated by this model (Ezui et al., 2018). GUMCAS and CSM-CROPSIM-Cassava reduce fibrous root growth as the crop ages, while the CSM-MANIHOT-Cassava maintains a fixed partitioning rate of 10 % for aboveground growth. These models also consider specific root length to estimate root length density, which is used to calculate root water uptake. Gray (2003) also uses the root length density to estimate the plant water potential affecting transpiration, photosynthesis and leaf appearance; in this model the rooting depth defines the volume of soil that can be used for fibrous root growth. After supplying the demand for leaves and stems, SIMCAS allocates the additional assimilates to the fibrous roots before the storage roots initiate growth; Santhosh Mithra et al. (2013) did not specify the root distribution in the soil profile and how it affects the water and nutrient uptake.

In the model of Gutierrez et al. (1988b), N uptake depends on the total amount of N that is available in the root volume and N demand of the plant, which is proportional to the assimilates demand. The CSM models of DSSAT, i.e., CROPSIM and MANIHOT, follow a similar methodology using the root length density and N available in each layer to define the potential root N uptake. HyCAS estimates N uptake similar to Gutierrez et al. (1988b), although the demand is defined as the requirement to maintain N content in the organs. A similar approach is followed by LINTUL for N, P and K, and by SIMCAS for N and K. However, the availability of nutrients in those models is not defined by the root volume but instead by the entire supply of nutrients already available in the soil and from fertilizer applications. The LINTUL and CSM models of DSSAT also modify the demand of nutrients based on thermal age and the maximum and minimum concentration of the nutrients in the different organs (Adiele, 2020).

When available N is less than N demand, the N stress factor reduces growth demand in the model developed by Gutierrez et al. (1988b). The CSM models of DSSAT follow a similar approach, although CROPSIM reduces leaf expansion and radiation use efficiency while MANIHOT just affects leaf formation and expansion. LINTUL considers that there is nutrient stress when the nutrient concentrations are below the optimum value that is required for maximum growth. Because LINTUL considers N, P and K, the nutrient stress is defined as the product of each individual nutrient stress factor, which affects assimilate production and thus crop growth. High tree densities in the HyCAS model increases the competition for water and N and thus reduces cassava growth. The N and K stress factors of SIMCAS have a direct effect on the potential yield. Fukai and Hammer (1987) do not consider N uptake, but instead define a fertility factor that affects the partitioning to the aboveground organs with higher N promoting shoot growth. The QUEFTS models do not simulate the dynamic process of nutrient uptake, but simple calculations of nutrient uptakes are made based on the amount of N, P and K supplies (soil and inputs) and their interactions. Yield is then modified based on the amount of nutrient uptakes and the nutrient use efficiency of the cassava plant.

The two ‘oldest’ models (1, 15) do not consider solar radiation as an input. The models that account for respiration as part of the plant carbon simulations and the different partitioning strategies of the models are defined on Table 1. An example for the dynamic partitioning over time to aboveground biomass and root biomass is shown in Fig. 1b for GUMCAS, one of the twelve models that simulate the soil and plant water dynamics. Only six models consider the effect of VPD on stomatal closure, photosynthesis and transpiration (6, 7, 9–11, 14).

All cassava models simulate dry weight of the storage roots except for the SIMCAS model and the FAO Agroecological zone approach, which show model performance based on fresh biomass using a fixed ratio between dry and fresh weight (Santhosh Mithra et al., 2013; Visses et al., 2018).

## 5 Discussion

All cassava models that were reviewed in this study tend to provide accurate predictions of yield for the environments and for the limited number of genotypes for which they were originally created. Most of the model developers recognize that their models may have limitations in scope for application outside the areas for which they were developed and that they should only be used for the cultivars for which they were calibrated.

The models based on the development of the phytomers are more process-based than the other dynamic models. This is because these models simulate processes involved in individual phytomer development and their interactions both within the plant and with the external environment. These phytomer-based models have the potential advantage that they can be extrapolated to novel circumstances with a greater degree of confidence than other models when the range of variation of each individual parameter in the new circumstances is within the range used to develop the model. Thus, for example, if the processes in the model have separately been developed for cool temperatures and large vapor pressure deficits, even though no field data are available for the combination of hot summers with large vapor pressure deficits and cool winter temperatures, the model could simulate these conditions reasonably well. If on the other hand, frosts occur in the new circumstances and the processes do not consider this variable, the model is unlikely to be effective under these novel conditions.

A potential drawback for the more complex phytomer-based dynamic models is that they are constructed with many variables, parameters, and processes. Consequently, small inaccuracies in the simulation of the multiple processes can accumulate, leading to large errors in the final estimation of, for example, yield. The variation between simulated and actual yield of two of these complex models (CSM-CROPSIM-Cassava and CSM-MANIHOT-Cassava) was large when evaluated over a wide range of conditions (see blue symbols in Fig. 1a).

When accuracy of prediction is required, for example for planning based on end-of-season yield, the static and simpler dynamic models may be the most appropriate. However, when models are needed to guide future decision on how cassava will grow in unexplored environments, for new management scenarios, or how novel phenotypes will perform, the more complex dynamic models are appropriate. Furthermore, the more complex models offer the opportunity to do experiments *in silico* for unknown scenarios such as future climates with increased carbon dioxide levels. Additionally, these models can pinpoint gaps in our knowledge of crop development and to understand the interactions between distinct processes. The two models which assessed interaction between plants and pests illustrate how they can appraise interactions that are difficult to trial in the real world (Cock, 1978; Gutierrez et al., 1999).

There is scope for improving the dynamic phenological models to improve the correspondence between simulated and real events, but also to enhance their potential uses. Regarding the latter, we note that a major deficiency in all but one of the models is their inability to simulate the influence of increasing CO_2_ levels which are likely to markedly influence both carbon assimilation and transpiration. We propose specific improvements that could be made considering the idiosyncrasies of cassava. These quirks are laid out within the overall framework of the main drivers of yield: (i) development capacity to produce assimilate (leaf area and leaf disposition), (ii) radiation use efficiency for biomass production and (iii) the partition of assimilate. These drivers are analyzed with reference principally to the weather and soil conditions.

### 5.1 Leaf area and disposition

The validity of a crop model is largely defined by proper simulation of the dynamics of LAI during the growing season (Gabriel et al., 2014). The originators of the dynamic models recognize the difficulty of simulating leaf formation and expansion (Matthews and Hunt, 1994). Cassava, unlike many crops, markedly reduces its leaf area and maintains the leaf nutrient contents when the availability of nitrogen and phosphorous are limited (Cock, 1984; Cock and Connor, 2021). The Gutierrez model (Gutierrez et al., 1988b) and the recent version of the CSM-MANIHOT-Cassava model restrict leaf area development according to nitrogen availability and maintain the nitrogen content of the leaves. The incorporation of nutrient restrictions on top growth would likely remove the need for the arbitrary restrictions on branching and leaf growth in various models and, hence, should improve the models’ ability to accurately simulate LAI.

Model development is also required to account for VPD effects on crop growth. The minimal differences between leaf water potential of soil water stressed and unstressed plants during the day resulting from the large stomatal response to VPD suggest that soil water stress and VPD largely influence crop growth through reduced assimilate and nutrient availability as opposed to direct effects of water potential on leaf expansion.

Long days are known to accelerate flower initiation and the resulting forked branching typical of cassava. Although it has been suggested that photoperiod may affect leaf production rates per apex and leaf size, these appear to be indirect effects probably due to competition for plant nutrients (Cock and Connor, 2021). This hypothesis, if confirmed, should be included in the models with photoperiod only directly influencing branching habit. All the current models base photoperiod on data from just two Australian varieties, and hence more quantitative information is required on the sensitivity of distinct varieties to photoperiod at distinct growth stages. We stress accurate simulation of photoperiod effects as cassava is likely to expand into higher latitudes with global warming.

### 5.2 Biomass production

All the dynamic models base the production of biomass on the solar radiation intercepted by the leaves. The early models related crop growth rate directly to LAI. This simple model, *a priori*, appears unsatisfactory as it does not consider variation in solar radiation. However, a recent overview of cassava physiology indicated that the maximum crop growth rates for cassava at LAI > 4 is relatively constant at around 22−24 g m^−1^ d^−1^ over a wide range of conditions and is less than that of other crops with similar photosynthetic rates (Cock and Connor, 2021). This almost constant maximum crop growth rate fits with the observations that during periods of high solar radiation photosynthesis of cassava is restricted by VPD rather than solar radiation (Vongcharoen et al., 2019). Thus, crop growth rate as a function of LAI, if corrected for temperature and soil water stress, may serendipitously incorporate the combined effects of VPD and solar radiation.

The value for RUE or PARUE used in many of the models is determined from the biomass generated per unit of total (R) or photosynthetically active radiation (PAR) intercepted over a relatively long period. Moreover, RUE fluctuates significantly during the growing period (Adiele, 2020). Due to the VPD effect on photosynthesis, the potential RUE estimated from periods that include sunny, low air humidity periods will be underestimated. Hence, RUE may underestimate biomass produced under humid conditions. The higher-than-normal PARUE of 2.8 g MJ^−1^ with generally high humidity and little seasonal water stress (Adiele et al., 2021) suggests that potential RUE values obtained from high humidity conditions should be used.

Several models include a VPD factor to modify RUE. As the VPD effect is via partial stomatal closure, it will also modify transpiration in the models. In all the models that have a correction factor for the VPD effect, except DYNCAS, a daily average VPD is used. As the VPD effect varies throughout the day, we recommend the hourly estimates as in the DYNCAS and maize models (Messina et al., 2015). The incorporation of a factor for the VPD effect on stomatal conductance suggests that RUE values obtained under high humidity conditions, should be used when correction is made for the VPD effect. However, without correction average RUE values may be better. As stated above, gas exchange is also greatly affected by atmospheric CO_2_ concentrations, an aspect that despite its importance is only simulated by a revised SIMANIHOT model (Tironi et al., 2017b).

Several of the models incorporate maintenance respiration, a concept originally borrowed from animal science. The DYNCAS model considers the strategy indicated by Thornley (2011) and Cock and Connor (2021) to ignore maintenance respiration and ascribe respiration to growth processes. This is probably a safer path than the heroic assumptions made for maintenance respiration.

Gutierrez et al. (1988b) observed that their model was improved when limited nitrogen availability reduced overall growth rather than reducing photosynthesis. We suggest that the approach followed in the Gutierrez model fits with the generally observed reduction of plant size with minimal effects on photosynthetic rate with moderate nutrient deficiency (Cock and Connor, 2021).

### 5.3 Biomass distribution

Connor (2019) highlights three questions related to assimilate partitioning raised by Gray (2000). Is the development and growth of tubers affected by the assimilate demand of the stems? Is tuber growth limited by the sink capacity? Is the development and growth of the stems affected by the strength of tuber demand? The DYNCAS model addresses these questions and partitions available assimilate according to a maximum specific uptake rate for each organ at a saturating concentration and a Michaelis constant. However, conceptually a certain proportion of the assimilates could be directed towards labile reserves and later be remobilized. Depending on the constants used for the distinct organs, the DYNCAS approach can move between the rigid constant fractions used in some of the models and the fixed spillover model. Furthermore, this method lends itself to remobilization of reserves either from the stem or the tubers if part of stored reserves are part of the available assimilate.

### 5.4 Starch content

The starch or dry matter content of cassava is a critical determinant of crop quality. Farmers are often paid according to starch content or the cooking quality of the roots. None of the current models adequately handles starch or dry matter content, which is a major deficiency which needs to be remedied in future models.

## 6 Conclusions

The cassava modeling literature is relatively extensive, covering 18 models with a varying range of capabilities. In general, our review shows that these models perform well at predicting harvest yield, but that no single model covers the full range of processes that influence cassava growth. In addition, models have been generally tested in a limited range of environments compared to the range of environments in which cassava is cultivated. We, thus, conclude that all models are limited in their application domain, and that crop model improvement and more extensive testing are likely warranted if cassava models are to be used more widely.

Areas of special emphasis include yield responses to management practices, (e.g., intercropping, combined N, P, K responses), biotic factors, photoperiod, vapor pressure deficit, and climate change (especially CO_2_ response), as well as the simulation of both accumulation and remobilization of carbohydrate reserves and, of paramount importance, tuber quality. Due to their dynamic nature, process-based models that have been developed handling genetic or varietal differences and are based on multiple environments that consider weather and soil variables have the greatest potential for improvement. However, new experimental work will likely be needed for crop model design, parameterization, and evaluation. If these data and model limitations are addressed, the promise and potential of cassava crop models to be used to inform farming and policy decisions can be realized.

## Declaration of Competing Interest

The authors declare no conflict of interest.
